# The Maxilla-Mandibular Discrepancies through Soft-Tissue References: Reliability and Validation of the Anteroposterior Measurement

**DOI:** 10.3390/children10030459

**Published:** 2023-02-26

**Authors:** Cinzia Maspero, Niccolò Cenzato, Francesco Inchingolo, Maria Grazia Cagetti, Gaetano Isola, Davide Sozzi, Massimo Del Fabbro, Gianluca Martino Tartaglia

**Affiliations:** 1 Department of Biomedical, Surgical and Dental Sciences, University of Milan, 20122 Milan, Italy; 2 Fondazione IRCCS Cà Granda, Ospedale Maggiore Policlinico, 20122 Milan, Italy; 3 Department of Interdisciplinary Medicine, Università degli Studi di Bari “Aldo Moro”, 70124 Bari, Italy; 4 Department of General Surgery and Surgical-Medical Specialties, School of Dentistry, University of Catania, 95124 Catania, Italy; 5 Department of Maxillofacial Surgery, Fondazione IRCCS San Gerardo dei Tintori, School of Medicine and Surgery, University of Milano-Bicocca, 20900 Monza, Italy

**Keywords:** Wits appraisal, orthodontic discrepancy, Angle’s malocclusions skeletal classification, cephalometric diagnosis

## Abstract

This research aimed to identify a new measurement to diagnose the sagittal maxillary and mandibular difference that considers the patient’s profile (soft tissue Wits appraisal: obtained by projecting points A and B on the occlusal plane and subsequently measuring the linear distance between the two points). This new measurement was compared to the conventional Wits appraisal obtained to diagnose sagittal jaw discrepancy. In total, 300 subjects (162 males, 138 females) aged between 6 and 50 years requiring orthodontic treatment were analyzed. The cephalometric tracings on the pre-treatment lateral cephalometric radiographs were obtained and the two measurements taken were the Wits appraisal and a new measurement which were both calculated and compared. The analysis of the linear correlations between the conventional Wits value and the measurement obtained on the soft-tissue were undertaken. The relevance was established at 5% (*p <* 0.05). The mean values were also calculated within sex and age classes, and comparisons between sexes were obtained employing the t test Student’s for independent samples. Subsequently, chi-square analysis was also utilized to verify the sex distributions in the age groups considered. The results obtained suggested that these two measurements were significantly correlated with each other, with no characteristic patterns of sex or age. These data allowed an estimation of the reference values of the new measurement (−1.9 mm and 5.4 mm for patients with Class I molar relationship) showing that the former was more variable than the latter. The new measurement could allow for an accurate evaluation of the jaws sagittal discrepancy from soft tissue analysis.

## 1. Introduction

The assessment of the magnitude of the maxillary and mandibular sagittal discrepancy is essential in orthodontic diagnosis and for the evaluation of facial proportions [1,2,3].

Various linear and angular cephalometric measurements have been proposed, but none of them appears to give reliable and easy significative clinical analysis [4]. Cephalometry is considered for both clinical prediction and growth planning [2]. The ANB angle obtained from the subspinal-nasion and supramental points and the “Wits appraisal” obtained by projecting the A and B points onto the occlusal plane and considering the linear distance between them, are the most used and described measurements in the literature [5,6,7].

However, both these methods have several shortcomings from a clinical point of view [6,8,9]. Firstly, the SNA (sella-nasion-point A) and ANB (A-nasion-point B) angles are easily affected by slight modifications in the location of the upper and lower jaws with regard to the cranial base. Moreover, the Wits appraisal has been queried because of the difficulties in recognizing the occlusal functional plane and its change in inclination [6]. As such, other options have been proposed over the years that are based neither on the cranial base nor the occlusal plane [5,9]. Indeed, both measurements evaluate the positions of the maxilla and the mandible relative to each other on the sagittal plane, but while the ANB angle depends also on the relative locations of the cranial base and jaws, the Wits appraisal measures the sagittal jaw relationship independently to cranial landmarks [5,6,10].

To overcome these limitations, new cephalometric measurements have been proposed to increase diagnostic precision [11]. Hall-Scott and co-workers [12] proposed to project A and B points onto the bisector of the intermaxillary angle, and to quantify this sagittal discrepancy. Nanda and Merrill (1994) with others suggested using the projection of the A and B points on the palatal plane and measuring this discrepancy [13,14]. In 1995, Yang and Suhr rated the angle between the Frankfurt horizontal plane and the one obtained joining points A and B and, in 1999, Ferrario et al., recommended that the diagnosis of anteroposterior jaws discrepancies should be obtained by considering more than a single anteroposterior appraisal [3,15]. All these measurements clearly yielded good results in the original samples, however, none of them took into consideration the patient’s soft tissues.

A frequent complaint of patients concerns the overall appearance of the face, which results from the combined contributions of the hard and soft tissues. Therefore, the diagnosis should give due importance to both the soft tissues and the skeletal structures. [15]

Sagittal upper and lower jaw relationships have been assessed using several parameters such as the facial and the angle of convexity, the A–B plane angle, the APDI, and the AF–BF angle [16,17,18].

Points A and B have the disadvantage to be on the anterior limitations on the alveolar bone and they can be affected by orthodontic treatment. Facial analysis contours can identify and localize areas of disproportion and compensation. According to Worms and colleagues, the major compensating factors are the facial soft tissues, as such a detailed diagnosis and treatment planning should take into careful account a meaningful analysis of facial contour [19].

Many authors have reported that in order to have successful treatment results, facial soft tissues should be careful evaluated [20].

In 2021, Sreenivasagan proposed to assess on the lateral teleradiographies the sagittal relationship of the soft tissue while considering the skeletal points and the cranial reference planes based on the soft tissue landmarks [13]. Patients are aware of the changes in their facial appearance, and as such, treatment plans based only on the cephalometric evaluation may not provide optimal results to the face. Being able to understand soft tissue adaptations is important in order to achieve aesthetic results [21].

Furthermore, the soft tissue profile can help in making therapeutic decisions - such as the need for advancing the maxilla or the mandible or teeth extractions. Some authors have proposed the soft tissue plane to upper incisor (UI), the Barcelona line, to identify the best anteroposterior location of the upper jaw [22]. E.M. Floyd, S.W. Perkins and since 1983 Reed A. Holdaway have demonstrated the insufficiency of hard-tissue analysis alone for the diagnosis and treatment planning for soft-tissue analysis [23,24,25].

Subsequently, the aim of this research was to identify a new sagittal measurement of maxillo-mandibular discrepancies taking into account the soft tissue. The methods described in this study included a measurement of the anteroposterior upper and lower jaws discrepancy to diagnose Angle’s skeletal Class malocclusion. This should assist in providing a complete morphological study of the skeletal Class by analyzing soft tissues of the facial surface in an accurate, reproducible way. Furthermore, the study aimed to verify the correlation of this new measurement with an established linear assessment of the anteroposterior discrepancy, the Wits appraisal. This correlation also allowed an estimation of the reference values of the measurement.

## 2. Materials and Methods

Fondazione IRCCS Ca Granda, Ospedale Maggiore, Milan, Italy provided the ethical approval for this study (9 March 2016; n. 421) and the protocol was planned in agreement with the principles of the Declaration of Helsinki adopted for medical studies including humans, including all amendments and revisions. A written informed consent for all the procedures described below and for the use of data collected from their medical records for scientific purposes was signed by all the patients/parents/caregivers.

### 2.1. Sample

In total, 750 images taken from January 2012 to June 2016 at the Complex Unit of Dentistry and Maxillo-Facial Surgery at the Policlinico of Milan and the first 300 images of patients meeting the inclusion criteria were selected. Only 300 images were included because they comprised the characteristics that prospective research participants were required to have in order to be included in the study. The data used were extrapolated from the medical records of patients.

All lateral cephalograms to be included in the sample had to belong to patients with the following characteristics:

1. Caucasian subjects with the same skeletal and dental patterns were selected in order to identify reliable landmarks and to subsequently extend to other ethnic groups;
2. subjects with dental and skeletal malocclusions such as Class II or III, crowding, dental malpositions;
3. mixed or permanent dentition;
4. subjects who showed 3 mm of maximum difference between the right and the left Gonion and Maxillary points from the midsagittal plane in the frontal cephalometric analysis according to Hwang et al., 2007 [13];
5. no cross-bite;
6. no patients with surgical needs were evaluated.

The exclusion criteria were:

1. the absence of molars or premolars;
2. past or current orthodontic therapies;
3. bone metabolism modifications;
4. the presence of asymmetry greater than 2 mm between the right and left homologous cephalometric landmarks;
5. maxillofacial skeleton discrepancies (acquired or congenital).

The reason for the selection of subjects in need of orthodontic treatment as the sample for this study was primarily ethical, as it is not possible to expose subjects to ionizing radiation if they do not need it.

The images analyzed in the study were acquired with the I-CAT FLX (Imaging Sciences International, Hatfield, PA, USA) scanner, which was used for all the images. The scanning procedure implicated a slice of 4 mm thickness, a field of view of 16 × 22 cm, a scan time of 20-s, and a voxel size of 0.49/0.49/0.5 mm.

**Figure 1:**
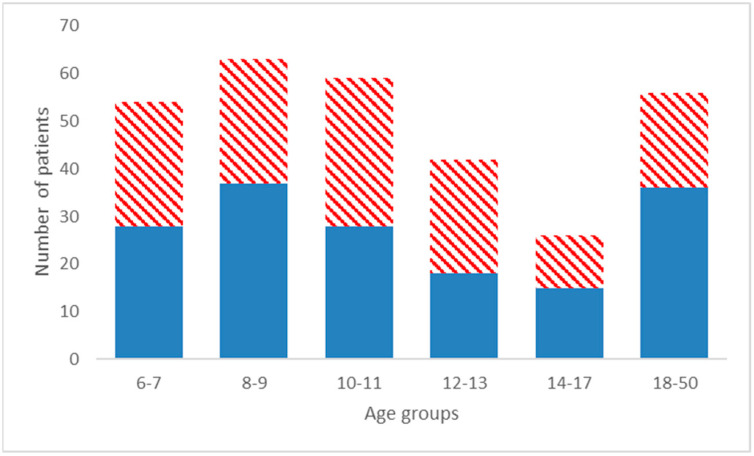
Distribution of the sample by sex and age class. Ages, rounded to the nearest 6 months, are in years; filled blues columns are males; dashed red columns are females.

Three-hundred lateral cephalograms obtained before treatment of 162 males and 138 females patients age ranging between 6 and 50 years were selected. The patients were divided into six non-overlapping age groups (Figure 1).

### 2.2. Measurements

Raw data obtained from the CBCT scan were coded in the file format Digital Imaging and Communications in Medicine (Dicom3). Lateral cephalometric x-rays of the whole volume employing the software 1.8 iCAT Vision (Imaging Sciences International, Inc., Milan, Italy; https://ct-dent.co.uk/i-cat-vision/ (accessed on 15 April 2021)) were reconstructed for each raw data according to Baldini et al. (2022) [5].

**Figure 2:**
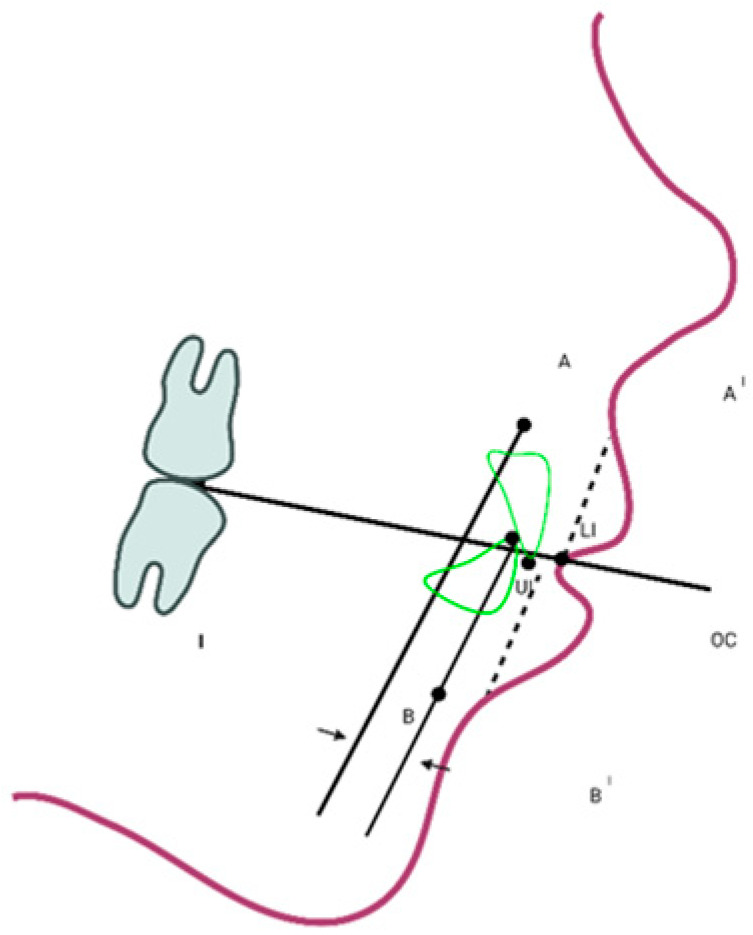
Digitized landmarks, and linear measurements acquired on the lateral teleradiographies. A prime indicates the equivalent of a hard-tissue landmark obtained on the soft-tissue. A–A’: subspinal point; B–B’: supramental point; LI: lower incisor; UI: upper incisor; Oc: point of occlusion between upper and lower permanet molars. The inserts indicate the two linear measurements obtained: I: conventional Wits appraisal; II: soft-tissue Wits appraisal (obtained projecting points A’ and B’ on the occlusal plane and then measuring the distance between them).

The 2D cephalometric x-rays were then digitized by the Dolphin Imaging Cephalometric and Tracing Software, version 11.9, Chatsworth, CA, USA, https://www.dolphinimaging.com/Media/DolphinNews?Subcategory_OS_Safe_Name=20160913 (accessed on 15 April 2021) from two expert orthodontists (NC, CM). Cephalometric landmarks were firstly recognized on the CBCT scans in the axial, coronal or sagittal plane and then verified in the other two planes and in the 3D volumetric rendering (Figure 2). New measurements and linear and angular traditional ones, were acquired according to Farronato et al. [6].

**Figure 3:**
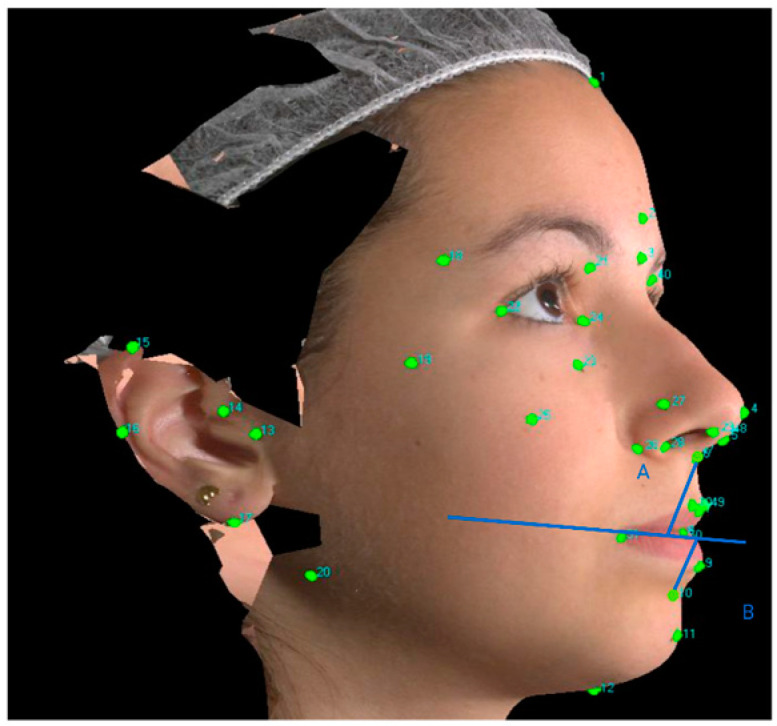
Soft tissue Wits appraisal.

The analyzed measurements identified and studied were the following:

1. Wits appraisal (obtained by projecting points A and B on the occlusal plane and subsequently measuring the distance between them, i.e., the plane drawn through the occlusal surfaces of the molar and the incisors), following the Hall-Scott procedure [15]
2. Soft-tissue Wits appraisal (obtained by projecting points A’ and B’ on the occlusal plane and then measuring the distance between them (soft tissue A’: the most inner point on the profile of the upper lip between the Subnasale and the upper lip anterior point; soft tissue B’: the most inner point on the contour of the mandibular sulcus contour). See Figure 3.

It should be noted that the soft tissue Wits appraisal, which was introduced in this study, did not include any identification or manual calculation (on the printout) of new reference, distances, or projection points. Therefore, it was not possible to separately assess the error of the single measurement from the global one (point, tracing and digitization) as described in the section “Error of method”.

Between the conventional Wits value and the one on the soft-tissue, linear correlations analyses were obtained. The significance was located at 5% (*p* < 0.05). The mean values were also calculated within sex and age class, and comparisons between sexes were carried out employing t test Student for independent samples. Furthermore, to test the sex distributions in the analyzed age groups a chi-square test was also utilized.

The objective of this investigation was to introduce a novel anteroposterior measurement technique for maxillo-mandibular discrepancies that considers the soft tissue. Additionally, the study sought to determine the correlation between the new measurement and the Wits appraisal, a commonly used linear evaluation of anteroposterior discrepancy. As a result of this correlation, the values of reference for the new measurement have been established. The Wits appraisal values for class II division I was considered to be 1.2 ± 3.3 as compared to class I −2.8 ± 3.3. Therefore, using the test comparing one mean of reference value at alpha significance of 0.05, 0.8% power, with a delta difference of −0.2 (difference between null value 1.2 mean and alternative value 1.0) at a 1.0 common standard deviation (SD), the total estimated sample size was considered to be *n* = 199. To prevent the attrition in the lateral cephalograms data availability, an additional 20% increase in the sample size was included.

### 2.3. Error of Method

The intra- and inter-operator reliability of the considered cephalometric measurements (ANB and AFBF) were reported in a previous paper [6]. In brief, three independent observers with the same background and 5 years of orthodontic practice executed three times cephalometric racings with a 15-day interval. ICC estimates for both intra-rater and inter-rater reliability and their 95% confidence intervals were calculated by the use of the SPSS^®^ 25.00 for Windows-based on single-measurement, absolute-agreement, 2-way mixed-effects model for each variable. Based on this source of error, for the current calculations two expert operators were calibrated with a training session in which the inter-examiner agreement for the examined characteristics was set at 95%.

Moreover, the same investigator digitalized again a sample of 30 images chosen randomly after 30 days. Each set of cephalometric points was normalized regarding rotation and translation as follows: the x-axis was normalized aligning it on the Frankfurt plane (porion-orbitale) and the origin of the axes was then considered the centroid of the coordinates. Each pair of repetitions was then compared on a point-to-point base. The same traces repeated twice showed differences of up to 2 mm (average, 1.2 mm), while the tracing of the same repeated radiographs showed differences of up to 2.5 mm (average, 1.8 mm).

## 3. Results

Three-hundred lateral cephalograms obtained before the treatment of 162 males and 138 females patients (age 6 to 50 years) were selected. The patients were divided into six non-overlapping age groups. Figure 1 shows the number of male and female patients in each age group.

Conventional and soft-tissue Wits appraisals showed no remarkable female-male differences in the six age groups considered (*p* > 0.05 for all tests). Furthermore, the age distribution (Figure 1) was found not to be sex-dependent (Chi-square test, *p* > 0.05). Thus, all subsequent calculations were obtained on the collective males plus females sample.

The analysis of the sagittal lower and upper jaws discrepancy of the two measurements (conventional and soft tissue Wits appraisal) is shown in Table 1 (mean values, standard deviations, and range). For both the variables, a wide range was identified in all age groups, in addition to a high standard deviation that was greater than the relative mean in almost all cases. The variability was greater for the conventional Wits appraisal than for the soft-tissue analysis. No age-related pattern was identified for the considered distances. Indeed, this result confirmed the heterogeneous constitution of the considered unselected sample, in which individuals of all dental and/or skeletal classes were included.

The soft tissue and conventional Wits appraisals showed significant correlations (*p* < 0.001) in all age groups, with linear correlation coefficients ranging between 0.719 (adolescents aged 14–17 years) and 0.880 (adults ages 18–50 years). This means that at least 57% of the variance of the conventional hard-tissue measurement could be explained by the soft-tissue assessment.

All obtained correlations suggested no age patterns, i.e., in all age groups a similar slope of the correlation line and its intercept were observed. In addition, an age-independent analysis was then performed, and a correlation line was calculated on the total sample (300 images): soft-tissue Wits (mm) = 0.877 × conventional Wits (mm) + 3.644. The relevant correlation coefficient was 0.817, which means that approximately 67% of the variance of one measurement can be predicted from the other. This correlation permits an evaluation of the “normality” of the soft-tissue Wits value corresponding to upper and lower limits. Indeed, considering that individuals with Class I skeletal relationship should present Wits appraisals comprised among −2 + 2 mm, and taking into account the standard errors of the intercept (SE = 0.0257) and slope (SE = 0.0071) of the present correlation line, the limits of normality of the soft-tissue Wits values should be −1.86 mm (lower limit, between patients with diagnosis of Class I and Class III) and 5.42 mm (upper limit, between patients with diagnosis of Class I and Class II).

## 4. Discussion

Traditional evaluation of the sagittal discrepancy between the maxilla and the mandible have been generally performed on the bone structure without considering the soft-tissue profile [6,7,26,27]. Indeed, while the underling upper and lower jaws notify most of the facial shape, the real physiognomy of the considered patient is due also to soft tissues [28,29,30].

Maxillofacial surgeons and orthodontists have always been attentive in identifying and comprising the morphology of the face in its totally, i.e., considering the soft-tissue drape and the underlying hard-tissue. When X-ray cephalometry was introduced in 1931 [31,32], however, the focus changed from the analysis of the overall face to the hard-tissue underlying it; in the next decades conventional cephalometric analysis studied only skeletal structures because it was thought was that the morphology of the soft-tissue was primarily linked to the morphology of the skeletal configuration. Since the early 1960s it was demonstrated that a close connection between the soft and skeletal tissues could exist. Since then, there has been an emphasis on the importance of a better estimation of soft tissue draping [16,17]. Despite the overwhelming scientific evidence, usually treatment planning considers the normal value detected on the lateral cephalometric tracings in two-dimension, ignoring the soft tissue morphology. Cephalometry on lateral teleradiograph has been taken as the diagnostic exam to determine the reference values to establish the patient’s skeletal pattern, but currently maxillofacial surgeons and orthodontists are inclined to study and rely more on soft tissue assessment than cephalometric tracings when planning treatment [33,34].

Baik and Ververidou in 2004 described a method to diagnose the anteroposterior position of the jaws, the Beta angle, which does not consider dental or cranial landmarks and is identified between the A-B line and at one perpendicular from point A to C-B line (condylion to B point) [35,36,37]. Conventional 2D x-rays have greatly utilised to diagnose anteroposterior discrepancy with an interest in skeletal landmarks. Soft tissue analysis permits a vision of the craniofacial structures accurately and completely allowing orthodontists to create new reference planes. This is an important diagnostic evolution for more accurate measurements which will help to achieve better results [15,36]. In the present study, a conventional linear measurement of hard tissue, namely the Wits appraisal, was correlated with its soft tissue equivalent. This kind of procedure is not unusual for clinical [32] and/or cephalometric purposes [34].

The present soft tissue measurement projects two cutaneous landmarks on a dental reference plane, and it cannot be considered a fully “soft-tissue” evaluation. However, it permits a quantitative evaluation of the sagittal analysis of the position of the maxilla relative to the mandible that also considers soft tissue draping. Furthermore, this new measurement could also be obtained on a lateral photograph of the patient, where the occlusal plane was identified by a Fox plane positioned between the upper and lower teeth [34], opening the way for a mobile phone application approach for fast and reliable non-invasive measurements. This assessment does not correspond exactly to the bisecting occlusal plane used by the Wits appraisal, because it is more influenced by the posterior (molars) than by the anterior (incisors) teeth, but it is commonly used in prosthetics. It can also overcome one of the possible limitations of the Wits appraisal performed on radiographs, namely the presence of radiopaque material (stainless steel, large restorations or prostheses) on the dental landmarks.

The new assessment was more homogenous among the analyzed sample than the conventional Wits appraisal. In fact, a large variability for the anteroposterior Wits measurement was also reported by Oktay and Hurmerinta et al. [1,4]. Previous investigations also reported no sex-related differences [1,3]. In the present large sample of both sexes covering almost the entire age range of orthodontic patients, the measurement of soft tissue correlated well with the assessment of hard tissue, and the correlation was therefore employed to identify alternative values for the new measurement that could be taken into consideration. Limits of −1.86 mm and 5.42 mm were acquired for Class I skeletal relationship patients. Certainly, it is not possible to consider these reference values as “norms” as the population taken into consideration required orthodontic treatment and the study did not consider healthy individuals with physiologic skeletal and occlusal relationships [29]. Indeed, while the norms of the measurements were considered on patients with physiologic or even “excellent” occlusions, when the biological price paid by patients was still considered ethical, such a group cannot be replicated currently, and the estimation made here was considered the best possible approach.

It is possible to obtain the values of reference for the new measurements using a wide group of patients or calculating the measurements on a conventional collection of “normal” radiographs. In this study, the cephalometric tracings were obtained with the Bolton standards on the lateral head film (male-female average) between 6 and 50 years of age and conventional Wits appraisals (range −1.9 to 1.5 mm, mean −0.79 mm, SD 0.96) and the present “soft-tissue” Wits (range 1.2 to 4.8 mm, mean 3.48 mm, SD 1.16) were calculated. The measurements were then properly correlated, with r = 0.683 (*p* < 0.05). A further reference interval for the new measurement of the anteroposterior discrepancy was calculated and values of 2.8 (lower limit, between patients presenting Class I and Class III) and 4.2 mm (upper limit, between patients presenting Class I and Class II) were thus obtained.

It has long been discussed that cephalometric analysis is relevant for obtaining the best treatment plan and evaluating the patient’s follow up [38]. The reliable correlation of the soft and hard skeletal tissue analysis of the maxillo-mandibular anteroposterior relationships found in the present sample begs the question: can the analysis of the facial morphology replace, at least in part, skeletal considerations? The present results showed that the measurement of the new soft tissue Wits appraisal could be appropriate for standard diagnoses and treatment planning, limiting the use of invasive examinations (i.e., those obtained from three-dimensional CT scan reconstruction) in the initial evaluation of serious malocclusions requiring combined orthodontic and surgical treatment.

Orthodontic diagnosis usually uses dental, skeletal and cephalometric landmarks to analyze malocclusions and develop treatment plans. In this study, a new soft tissue assessment of Wits was presented and evaluated. This parameter may provide orthodontists with a useful diagnostic tool to plan treatment that also include facial aesthetics evaluation in addition to the skeletal ones. In fact, the proposed new Wits assessment highlights problems associated with facial soft tissue labial segments in a simple and non-invasive manner, contributing to the study of the facial aesthetics and profile.

Within the limitations of this study, it should be considered that the definition of facial contour aesthetics changes in different ethnic groups and sexes and it has also changed in different historical periods. Furthermore, it is important to consider that nowadays many patients resort to aesthetic treatments, such as botulinum toxin or hyaluronic acid injections, which can alter the original profile. It must also be emphasized that each individual has unique facial features, which therefore makes it difficult to standardize facial aesthetics.

## 5. Conclusions

In this study a new method to diagnose Angle’s skeletal Class on the soft tissue profile was introduced for the purpose of evaluation in a quantitative manner on a profile photograph. Orthodontic treatment should always consider the aesthetic characteristics of each individual in order to achieve an improvement in the facial profile, in addition to correcting the underlying dentoskeletal alterations. The soft tissue Wits appraisal proposed appeared to be a simple, individualized, and reproducible measurement to aesthetically diagnose sagittal maxillary and mandibular position for orthodontic treatment plan. Furthermore, it would assist in correcting faults of the lower facial profile and even in every single jaw area, providing an overall improvement in face aesthetics and harmony.

## Figures and Tables

**Table 1:** Range of mean values and standard deviations of the two Wits appraisals, and significant linear correlation coefficients.

Age	Wits (mm)			Soft-Tissue Wits (mm)			Correlation Coefficient
Group	Mean	SD	Range	Mean	SD	Range	X: Wits
(yrs)							Y: Soft-Tissue Wits
6–7	−0.61	3.92	−9.8–7.1	2.40	3.88	−6.9–9.7	0.876
8–9	0.61	2.83	−5.7–6.8	3.90	3.11	−7.5–10.3	0.752
10–11	0.52	2.79	−5.8–6.6	4.54	3.40	−3.7–11.0	0.786
12–13	0.22	3.14	−6.6–5.4	3.63	3.67	−5.7–10.6	0.796
14–17	0.90	3.49	−6.2–9.7	4.76	3.37	−3.5–11.5	0.719
18–50	0.09	5.20	−18.8–11.5	4.49	5.23	−9.8–14.9	0.880
Total	0.22	3.61	−18.8–11.5	3.83	3.88	−9.8–14.9	0.817

Wits: obtained projecting points A and B on the occlusal plane and then measuring the distance between them. “Soft-tissue” Wits: obtained projecting points A’ and B’ on the occlusal plane and then measuring the distance between them. All correlations are significant at the 0.001 level.

## Data Availability

The data presented in this study are available on request from the corresponding author. The data are not publicly available due to privacy law.

## References

[1] Oktay H. (1991). A comparison of ANB, WITS, AF-BF, and APDI measurements. Am. J. Orthod. Dentofac. Orthop..

[2] Moate S.J., Geenty J.P., Shen G., Darendeliler M.A. (2007). A new craniofacial diagnostic technique: The Sydney diagnostic system. Am. J. Orthod. Dentofac. Orthop..

[3] Yang S.D., Suhr C.H. (1995). F-H to AB plane angle (FABA) for assessment of anteroposterior jaw relationships. Angle Orthod..

[4] Hurmerinta K., Rahkamo A., Haavikko K. (1997). Comparison between cephalometric classification methods for sagittal jaw relationships. Eur. J. Oral Sci..

[5] Baldini B., Cavagnetto D., Baselli G., Sforza C., Tartaglia G.M. (2022). Cephalometric measurements performed on CBCT and reconstructed lateral cephalograms: A cross-sectional study providing a quantitative approach of differences and bias. BMC Oral Health.

[6] Farronato M., Maspero C., Abate A., Grippaudo C., Connelly S.T., Tartaglia G.M. (2020). 3D cephalometry on reduced FOV CBCT: Skeletal class assessment through AF-BF on Frankfurt plane—Validity and reliability through comparison with 2D measurements. Eur. Radiol..

[7] Farronato M., Baselli G., Baldini B., Favia G., Tartaglia G.M. (2022). 3D Cephalometric Normality Range: Auto Contractive Maps (ACM) Analysis in Selected Caucasian Skeletal Class I Age Groups. Bioengineering.

[8] Cenzato N., Iannotti L., Maspero C. (2021). Open bite and atypical swallowing: Orthodontic treatment, speech therapy or both? A literature review. Eur. J. Paediatr. Dent..

[9] Lee M., Kanavakis G., Miner R.M. (2015). Newly defined landmarks for a three-dimensionally based cephalometric analysis: A retrospective cone-beam computed tomography scan review. Angle Orthod..

[10] Zamora N., Cibrian R., Gandia J., Paredes V. (2013). Study between anb angle and Wits appraisal in cone beam computed tomography (CBCT). Med. Oral Patol. Oral Y Cir. Bucal.

[11] Kotuła J., Kuc A.E., Lis J., Kawala B., Sarul M. (2022). New Sagittal and Vertical Cephalometric Analysis Methods: A Systematic Review. Diagnostics.

[12] Hall-Scott J. (1994). The maxillary-mandibular planes angle (MM°) bisector: A new reference plane for anteroposterior measurement of the dental bases. Am. J. Orthod. Dentofac. Orthop..

[13] Sreenivasagan S., Sivakumar A. (2021). FSA Angle: A Soft Tissue Approach for Assessing Sagittal Skeletal Discrepancy. Int. J. Clin. Pediatr. Dent..

[14] McNamara J.A. (1984). A method of cephalometric evaluation. Am. J. Orthod..

[15] Ferrario V.F., Sforza C., Miani A., Tartaglia G.M. (1999). The use of linear and angular measurements of maxillo-mandibular anteroposterior discrepancies. Clin. Orthod. Res..

[16] Nanda R.S., Merrill R.M. (1994). Cephalometric assessment of sagittal relationship between maxilla and mandible. Am. J. Orthod. Dentofac. Orthop..

[17] Chang H.-P. (1987). Assessment of anteroposterior jaw relationship. Am. J. Orthod. Dentofac. Orthop..

[18] Kim Y.H., Vietas J.J. (1978). Anteroposterior dysplasia indicator: An adjunct to cephalometric differential diagnosis. Am. J. Orthod..

[19] Worms F.W., Isaacson R.J., Speidel T.M. (1976). Surgical orthodontic treatment planning: Profile analysis and mandibular surgery. Angle Orthod..

[20] Ackerman J.L., Proffit W.R., Sarver D.M. (1999). The emerging soft tissue paradigm in orthodontic diagnosis and treatment planning. Clin. Orthod. Res..

[21] Kasai K. (1998). Soft tissue adaptability to hard tissues in facial profiles. Am. J. Orthod. Dentofac. Orthop..

[22] Hernández-Alfaro F., Vivas-Castillo J., de Oliveira R.B., Hass-Junior O., Hernando M.G., Valls-Ontañón A. (2023). Barcelona line. A multicentre validation study of a facial projection reference in orthognathic surgery. Br. J. Oral Maxillofac. Surg..

[23] Floyd E., Perkins S. (2019). Anatomy of the facial profile. Facial Plast. Surg..

[24] Reed A. (1983). Holdaway. A soft-tissue cephalometric analysis and its use in orthodontic treatment planning. Part I. Am. J. Orthod..

[25] Budai M., Farkas L., Tompson B. (2003). Relation between anthropometric and cephalometric measurements and proportions of the face of healthy young white adult men and women. J. Craniofac. Surg..

[26] Almaqrami B.-S., Alhammadi M.-S., Cao B. (2018). Three dimensional reliability analyses of currently used methods for assessment of sagittal jaw discrepancy. J. Clin. Exp. Dent..

[27] Tumedei M., Piattelli A., Falco A., De Angelis F., Lorusso F., Di Carmine M., Iezzi G. (2020). An in vitro evaluation on polyurethane foam sheets of the insertion torque, removal torque values, and resonance frequency analysis (RFA) of a self-tapping threads and round apex implant. Cell. Polym..

[28] Buccino F., Zagra L., Savadori P., Galluzzo A., Colombo C., Grossi G., Banfi G., Vergani L.M. (2021). Mapping local mechanical properties of human healthy and osteoporotic femoral heads. Materialia.

[29] Broadbent B.H., Broadbent B.H., Golden W.H. (1975). Bolton Standards of Dentofacial Developmental Growth.

[30] Hwang H.-S., Youn I.-S., Lee K.-H., Lim H.-J. (2007). Classification of facial asymmetry by cluster analysis. Am. J. Orthod. Dentofac. Orthop..

[31] Ferrario V.F., Sforza C., Miani A., Tartaglia G. (1993). Craniofacial morphometry by photographic evaluations. Am. J. Orthod. Dentofac. Orthop..

[32] Masucci C., Franchi L., Franceschi D., Pierleoni F., Giuntini V. (2021). Post-pubertal effects of the Alt-RAMEC/FM and RME/FM protocols for the early treatment of Class III malocclusion: A retrospective controlled study. Eur. J. Orthod..

[33] Bittner C., Pancherz H. (1990). Facial morphology and malocclusions. Am. J. Orthod. Dentofac. Orthop..

[34] Ferrario V.F., Sforza C., Tartaglia G., Barbini E., Michielon G. (1995). New Television Technique for Natural Head and Body Posture Analysis. Cranio®.

[35] Baik C.Y., Ververidou M. (2004). A new approach of assessing sagittal discrepancies: The Beta angle. Am. J. Orthod. Dentofac. Orthop..

[36] Sherman S.L., Woods M., Nanda R.S., Currier G.F. (1988). The longitudinal effects of growth on the Wits appraisal. Am. J. Orthod. Dentofac. Orthop..

[37] Bishara S.E., Fahl J.A., Peterson L.C. (1983). Longitudinal changes in the ANB angle and Wits appraisal: Clinical implications. Am. J. Orthod..

[38] Zamora N., Llamas J., Cibrian R., Gandia J., Paredes V. (2012). A study on the reproducibility of cephalometric landmarks when undertaking a three-dimensional (3D) cephalometric analysis. Med. Oral Patol. Oral Y Cir. Bucal.

